# Epidemiology, risk factors, species distribution, and antifungal susceptibility of candidemia among hospitalized patients with COVID-19

**DOI:** 10.18502/cmm.7.4.8406

**Published:** 2021-12

**Authors:** Hasti Kamali Sarvestani, Shahram Mahmoudi, Pegah Afarinesh Khaki, Saham Ansari, Sara Ghaderkhani, Davoud Roostaei, Roshanak Daei Ghazvini, Seyed Jamal Hashemi, Zahra Rafat, Alireza Abollahi

**Affiliations:** 1 Department of Medical Parasitology and Mycology, School of Public Health, Tehran University of Medical Sciences, Tehran, Iran; 2 Department of Parasitology and Mycology, School of Medicine, Iran University of Medical Sciences, Tehran, Iran; 3 Department of Molecular Genetics, Faculty of Advanced Science and Technology, Islamic Azad University, Tehran, Iran; 4 Department of Medical Parasitology and Mycology, School of Medicine, Shahid Beheshti University of Medical Sciences, Tehran, Iran; 5 Department of Infectious Diseases, Imam Khomeini Hospital Complex, Tehran University of Medical Sciences, Tehran, Iran; 6 Department of Pharmacology, School of Medicine, Guilan University of Medical Sciences, Rasht, Iran; 7 Department of Medical Microbiology, School of Medicine, Guilan University of Medical Sciences, Rasht, Iran; 8 Department of Pathology, Imam Hospital Complex, Tehran University of Medical Sciences Tehran, Iran

**Keywords:** Candidemia, *C. albicans*, *C. dubliniensis*, COVID-19, Iran

## Abstract

**Background and Purpose:**

The pandemic of COVID-19 has caused a worldwide health crisis. Candidemia is a potentially lethal condition that has not yet been enough discussed in patients with COVID‐19. The current study aimed to investigate the prevalence of candidemia among Iranian COVID‐19 patients and characterize its causative agents and the antifungal susceptibility pattern

**Materials and Methods:**

The present cross-sectional survey was carried out from March 2020 to March 2021 at Imam Khomeini Hospital, Tehran, Iran. Blood specimens were obtained from patients with confirmed coronavirus infection who also had criteria for candidemia and were examined for any *Candida* species by conventional and molecular techniques. Susceptibility of isolates to amphotericin B, voriconazole, itraconazole, fluconazole, caspofungin, and 5-flucytosine was tested using the CLSI broth dilution technique.

**Results:**

In total, 153 patients with COVID-19 were included and candidemia was confirmed in 12 (7.8 %) of them. The majority of patients were ≥ 50 years of age (n=9) and female (n=8). Moreover, 6 out of the 12 patients were diabetic. The presence of central venous catheters, broad-spectrum antibiotic therapy, ICU admission, and mechanical ventilation was observed in all patients. The *C. albicans* (n=7, 58.3 %) and *C. dubliniensis* (n=2, 16.7%) were the most common isolated species. Amphotericin B and 5-flucytosine were the most active drugs. Despite antifungal treatment, 4 out of 12 patients (33.3 %) died.

**Conclusion:**

Due to the high mortality, the early diagnosis and proper treatment of candidemia are essential requirements for optimal clinical outcomes in COVID-19 patients.

## Introduction

The world is presently facing a major pandemic of coronavirus disease 2019 (COVID-19) originated at Wuhan, located in the Hubei province of central China, in early December 2019 [ 1 ]. The COVID-19 death rates vary significantly across different regions [ 2 ]. Until November 13, 2021, there have been a total of 127809 deaths related to this infection in Iran [ 3 ]. Among the major comorbidities in COVID-19 patients, insufficient consideration has been given to the incidence of fungal co-infections. Fungal co-infection with COVID-19 is critical especially in those that experience intubation, lymphocytopenia (a median lymphocyte count <1000 cells/mm^3^), hospitalization in intensive care units (ICUs), prolonged broad‐spectrum antibiotic usage, corticosteroid therapy, and immunocompromised individuals due to underlying conditions [ 2 , 8 ].

*Candida* species are opportunistic fungal pathogens that serve as part of the human normal microflora of mucosal and skin surfaces [ 4 - 6 , 9 ]. Due to indeterminate treatments for COVID-19, multi-drug treatment, invasive procedures, and uncertain and complex effects of the virus, *Candida* species can invade the bloodstream and cause candidemia, a life-threatening infection [ 7 ].

The global incidence of candidemia has increased in the last decade as a result of the change in the frequency of at-risk population, increase in the administration of broad-spectrum antibacterial agents, increase in the use of invasive methods (i.e., parenteral nutrition, peripheral vascular angioplasty, atherectomy, stents, mechanical ventilation, cardiac catheterization), use of complex surgical procedures, and immune-suppressive therapy [ 1 , 2 ]. These conditions are common among patients with infections caused by COVID-19.

Furthermore, the emergence of antifungal drug-resistant *Candida* isolates, especially pan-echinocandin-resistant *C. glabrata* [ 10 ] and multidrug-resistant *C. auris* [ 11 , 12 ], is a matter of concern. In a retrospective, multicenter study the epidemiology of COVID-19-associated candidemia in the ICUs of two COVID-19 centers in Mashhad, Iran was assessed. The results showed that among 1988 patients with COVID-19 admitted to ICUs, seven had fungemia [7/1988; 0.03%].

In total, nine yeast isolates (*C. albicans* [5/9;55%], *C. glabrata* [3/9; 33.4%], and *Rhodotorula mucilaginosa* [1/9; 11.1%]) were collected from patients with fungemia. Half of the patients infected with *C. albicans* (2/4) were refractory to both azoles and echinocandins. The mortality of patients with COVID-19-associated candidemia and patients with COVID-19 but without candidemia differed (100% [6/6] vs. 22.7% [452/1988]) [ 13 ]. In another study published by Arastefar et al., (Shiraz, Iran), 113 yeast isolates were recovered from 109 patients with candidemia, and the mortality rate was 28%. 

Almost 30% of the patients were from ICUs. *C. albicans* (56/113; 49.5%) was the most frequently involved pathogen followed by *C. glabrata* (26/113; 23%), *C. parapsilosis* (13/113; 11.5%), *C. tropicalis* (7/113; 6.2%), and *C. dubliniensis* (5/113; 4.4%). Five isolates showed antifungal resistance or decreased susceptibility to fluconazole: two *C. glabrata*, one *C. orthopsilosis*, one *C. albicans*, and one *C. dubliniensis* [ 14 ]. In Kashan, Iran, data from the Beheshti Educational Hospital showed that *C. albicans* (%50) was the most frequent causal agent of candidemia followed by *C. glabrata* (%40) and *C. parapsilosis* (%10). The mortality rate was high (%60) and most patients with candidemia were admitted to the ICU and neonatal ICU. All isolates were susceptible to amphotericin B, while the highest resistance belonged to caspofungin [ 15 ].

Until now, only one study has been published which addresses the epidemiology of candidemia and its mortality rate among Iranian COVID‐19 patients [ 13 ]. Hence, we conducted the present survey to provide much more detailed information about the epidemiology, risk factors, species distribution, and antifungal susceptibility of candidemia among the Iranian COVID‐19 patients.

## Materials and Methods

### Ethics statement

Informed consent was obtained from a person previously designated by the patient or a close family member (father, mother, children, aunts, uncles, grandparents, and cousins). Ethical approval for this study was obtained from the Ethics Committee of Tehran University of Medical Sciences (IR.TUMS.MEDICINE.REC.1399.366).

### Study population, sample collection, and clinical data

The present cross-sectional survey was carried out on a total of 153 patients hospitalized at Imam Khomeini Hospital Complex, a referral tertiary center in Tehran, Iran. All participants had positive real-time PCR tests for COVID-19 and also had at least one microbiologic and at least one clinical criterion for candidemia listed below [ 16 ], either at the time of study entry or within four days before the study entry.

(1) Microbiologic criteria:

• Candidemia (if one or more blood cultures were positive for *Candida* spp.)
• Other forms of invasive candidiasis (isolation of *Candida* spp. from at least one specimen obtained from a normally sterile site [other than blood] with or without a positive blood culture.)

(2) Clinical criteria:

• Fever in a patient who feels warm to the touch and/or an oral/tympanic temperature ≥ 100.4 °F (38.0 °C), and/or rectal temperature ≥101.4 °F (38.6 °C), and/or an axillary temperature ≥ 99.4 °F (37.4 °C).
• Signs or symptoms of candidemia/invasive candidiasis, which may include the following: feeding intolerance with increased gastric residuals, occult or gross bloody stools, distended stomach, color change, persistent and severe thrombocytopenia, lethargy, hyperglycemia, hypotension, glycosuria, and unexplained metabolic acidosis.
• To prevent false-negative results, patients who had taken any systemic antifungal agents before enrollment were excluded from the study. From March 2020 to March 2021, blood samples (approximately 20 mL for each patient) were collected aseptically via venipuncture after skin disinfection followed by 30 sec of drying [ 17 , 18 ]. The demographic and clinical data collected in this study were age, gender, underlying conditions, history of treatment with broad-spectrum antibiotics before candidemia, history of ICU admission before candidemia, presence of the central venous catheter, and mechanical ventilation at the onset of candidemia.

### Mycological examinations

Biphasic brain heart infusion media (Merck, Darmstadt, Germany) were utilized to culture blood samples. The blood culture vials were inoculated with blood specimens of patients, incubated at 37 °C for 7 days, and were tilted over a period of a half-hour every day and finally examined for the development of visible fungal colonies on the solid phase. Any growth of *Candida* species in blood cultures was considered as evidence of candidemia. Sabouraud dextrose agar (SDA) was the choice for the subculture of the recovered isolates, and typical *Candida* colonies were observed as cream/white-colored, pasty opaque slightly domed, and smooth appearance after 24-48 h at 37 °C on SDA. All isolates were confirmed using the molecular identification PCR- sequencing technique as below.

### Molecular technique

#### DNA Extraction

The genomic DNA was isolated from colonies grown on SDA using the high pure PCR templet purification kit (GeneAll Bldg, 303-7 105 Dongnam-ro, Songpa-gu, Seoul, South Korea) according to the recommended instructions of the manufacturer. 

### PCR analysis and sequencing

Each PCR reaction contained 2.5 μL of 10× reaction buffer, 2 mM MgCl, 500 μM dNTPs mixture, 1.25 µM Taq polymerase (Prime Taq, Genet Bio, South Korea), 25 pmol of each primer, 2 μL of DNA template solution, and enough distilled water to reach a final volume of 25 μL. In the present study, the amplification was conducted by the ITS1 (5′TCC GTA GGT GAA CCT GCG G 3′), and ITS4 (5′TCC TCC GCT TAT TGA TAT GC 3′) primers (Life Technologies, Barcelona, Spain). The PCR cycling conditions were as follows: initial denaturing at 95 °C for 5 min, followed by 35 cycles of denaturing at 94 ˚C for 30 s, annealing at 63 °C for 30 s, and elongation at 72 °C for 45 s. The final cycle was followed by an extension at 72 °C for 5 min.

Positive PCR products were examined by staining with DNA safe stain (4 µL) and electrophoresis on 1.5 % agarose gel. The PCR products were subjected to sequencing and analyzed using the MEGA7.0.21 software. All of the sequences had been deposited in GenBank under the accession number reported in Table 1.

**Table 1:** GenBank accession numbers of DNA sequences included in this study

Isolate	Molecular identification (ITS ^a^ gene)	GenBank accession number
SUB9504990 seq1	*C. tropicalis*	MW980762
SUB9504990 seq2	*C. albicans*	MW980763
SUB9504990 seq3	*C. albicans*	MW980764
SUB9504990 seq4	*P. kudriavzevii* (*C. krusei*)	MW980765
SUB9504990 seq5	*C. albicans*	MW980766
SUB9504990 seq6	*C. albicans*	MW980767
SUB9504990 seq7	*C. albicans*	MW980768
SUB9504990 seq8	*C. glabrata*	MW980769
SUB9504990 seq9	*C. dubliniensis*	MW980770
SUB9504990 seq10	*C. albicans*	MW980771
SUB9504990 seq11	*C. albicans*	MW980772
SUB9504990 seq12	*C. dubliniensis*	MW980763

ITS a: internal transcribed spacer

### Antifungal susceptibility testing

The Clinical and Laboratory Standards Institute document M27-A4 (CLSI M27-A4) broth microdilution protocol was used as a guideline for broth dilution method as an assay for testing antifungal susceptibility of yeasts [ 19 ]. Susceptibility of the isolates to fluconazole (Sigma Chemical Co, St Louis, Mo), amphotericin B (Bristol-Myers SP, Dublin, Ireland), flucytosine (Sigma Chemical Co), itraconazole (Janssen-Cilag, High Wycombe, UK), voriconazole (Pfizer Inc., New York, NY), and caspofungin (Merck Sharp &Dohme, Whitehouse Station, NJ) were tested. 

Stock and working concentrations of these drugs were prepared in 96-well microtiter plates based on the instructions stated by CLSI M27-A4 [ 19 ]. A suspension equivalent to 0.5 McFarland was prepared from an overnight yeast culture on SDA by suspending the fungal cells in 10 mL of sterile distilled water, and the concentration was spectrophotometrically adjusted according to CLSI M27-A4. Subsequently, 100 µL of fungal inocula were dispensed into wells of microplates, and results were read after 24 h of incubation at 35 °C. Two quality control (QC) strains, *C. parapsilosis* ATCC 22019 and *C. krusei* ATCC 6258 were also included. In the current study, interpretation of results was based on the breakpoints established for Candida species in CLSI- document M27-A4 (19).

### Statistical Analysis

Statistical analysis was performed with SPSS (version 24, IBM, Chicago, IL). We summarized the data by using descriptive statistics, presenting continuous variables as median, interquartile range, and categorical variables as proportions or percentages.

## Results

In the present study, during a period of 12 months (March 2020 to March 2021), 153 blood specimens were taken from 153 patients with coronavirus infection who also had criteria for candidemia. Of those, 12 (7.8 %) patients with candidemia were diagnosed. The mean age of patients was 57.5 years (age range of 32 to 85 years). The overall incidence of candidemia was higher in females than in males (rate ratio 2:1). The detailed information related to 12 patients with COVID-19-associated candidemia is shown in Table 2.

**Table 2:** Detailed information related to six patients with COVID-19-associated candidemia.

Variables		Patients	
		Number	Percentage
Age groups (years)	≥ 50	9	75
	< 50	3	25
Gender			
	Female	8	66.66
	Male	4	33.33
	Pneumonia	12	100
	Diabetes mellitus	6	50
	lymphocytopenia (500-900/mm^3^)	12	100
Underlying conditions and risk factors	Presence of central venous catheter at the onset of candidemia	12	100
	Broad-spectrum antibiotic therapy before candidemia	12	100
	Mechanical ventilation	12	100
	ICU admission prior to candidemia	12	100
Treatment with antifungals	Caspofungin alone	7	58.3
	Caspofungin + amphotericin B	3	25
	Voriconazole + caspofungin	2	16.7
Outcome	Died	4	33.33
	Alive	8	66.66

All the patients had pneumonia as an underlying condition, and one-half (6 of 12, 50%) of them were diabetic patients who developed candidemia in the ICU. The presence of the central venous catheter at the onset of candidemia, prior use of broad-spectrum antibiotics, mechanical ventilation, lymphocytopenia, and ICU admission before candidemia was observed in all patients with candidemia in the current study. 

According to the results of mycological and molecular methods, *C. albicans* (7 out of 12, 58.3%) was the predominant leading cause of candidemia followed by *C. dubliniensis* (2 out of 12, 16.6%), *C. tropicalis* (1 out of 12, 8.3%), *C. glabrata* (1 out of 12, 8.3%), and *Pichia kudriavzevii* (*C. krusei*) (1 out of 12, 8.3%). Antifungal therapy was administered to all patients; 7 of them were subjected to empirical monotherapy and 5 were treated by 2 antifungals. Despite antifungal therapy, 4 out of 12 patients who received antifungals (33.3%) died.

The results of susceptibility testing are presented in Table 3. Antifungal susceptibility test showed that Amphotericin B (MIC range: 0.06-1.0 µg/mL) and 5-flucytosine (MIC range: 0.03-4.0 µg/mL) were the most active drugs against all *Candida* isolates and no case of resistance to these antifungal agents was observed.

**Table 3:** In vitro antifungal susceptibility pattern of *Candida* isolates recovered from blood cultures of Iranian COVID-19 patients

Strains	Antifungals	MIC range	CLSI M27-A4 Breakpoints (n)	
			R	SDD /I
*C. albicans* (n=7)	AMB	0.06-1.0	0	0
	FLZ	0.06-64.0	1	0
	ITZ	0.015-0.25	0	1
	5FC	0.03-4.0	0	0
	VOR	0.015-0.5	0	1
	CAS	0.03-0.5	0	1
*C. dubliniensis* (n=2)	AMB	0.06-1.0	0	0
	FLZ	0.06-4.0	0	0
	ITZ	0.03-0.06	0	0
	5FC	0.03-1.0	0	0
	VOR	0.015-4.0	1	0
	CAS	0.03-0.5	0	1
*C. glabrata* (n=1)	AMB	0.125	0	0
	FLZ	64.0	1	0
	ITZ	0.03	0	0
	5FC	4.0.	0	0
	VOR	0.06	0	0
	CAS	2.0	1	0
*P. kudriavzevii* (*C. krusei*) (n=1)	AMB	0.5	0	0
	FLZ	64.0	1	0
	ITZ	0.03	0	0
	5FC	1.0	0	0
	VOR	1.0	0	1
	CAS	1.0	1	0
*C. tropicalis* (n=1)	AMB	0.5	0	0
	FLZ	1.0	0	0
	ITZ	0.03	0	0
	5FC	1.0	0	0
	VOR	0.03	0	0
	CAS	0.06	0	0

MIC: Minimum inhibitory concentration; R: resistant; SDD: susceptible-dependent-dose; I: intermediate; AMB: Amphotericin B; FLZ: Fluconazole; ITZ: itraconazole; 5FC: 5-flucytosine; VOR: voriconazole; CAS: caspofungin.

All the tested *Candida* isolates were found to be susceptible to fluconazole (MIC range: 0.06–64 µg/mL), except for one isolate of *C. albicans* (MIC:64 µg/mL), *C. glabrata* (MIC:64 µg/mL), and *Pichia kudriavzevii* (*C. krusei*) (MIC:64 µg/mL). Moreover, one isolate of *C. dubliniensis* (MIC: 4 µg/mL) was voriconazole-resistant, and one isolate of *C. albicans* (MIC:0.5 µg/mL) and *C. krusei* (MIC:1 µg/mL) showed intermediate resistance to this antifungal drug. On the other hand, one isolate of *C. krusei* (MIC: 1 µg/mL) was caspofungin-resistant, and one isolate of *C. albicans* (MIC:0.5 µg/mL) and *C. dubliniensis* (MIC:0.5 µg/mL) showed intermediate resistance to this drug. For itraconazole, 1 out of 7 (14.3 %) *C. albicans* isolates were classified as showing intermediate resistance, while the remaining isolates were susceptible to this antifungal.

## Discussion

The severe acute respiratory syndrome coronavirus-2 (SARS-CoV-2) caused a global pandemic burden with life-threatening outcomes [ 1 , 2 , 20 , 21 ]. Although global attempts (e.g., quarantine and lock-down) have been performed to overcome the pandemic, the incidence of COVID-19 infection and death is rising [ 22 ]. Fungal co-infections (especially invasive aspergillosis and mucormycosis) in patients with COVID-19 have been investigated in several studies [ 2 , 23 - 28 ]. Moreover, COVID-19 patients are prone to infections due to *Candida* species as a result of several risk factors. Mastrangelo et al. compared the incidence of candidemia in patients with SARS-COV2 infection to non–COVID-19 controls and concluded that the incidence rate of candidemia was significantly higher in patients with COVID-19 [ 29 ].

In the present study, candidemia was confirmed in 12 out of the 153 (7.8 %) COVID-19 patients. Similarly, a previous study reported an incidence of 8.9% for nosocomial candidemia in COVID-19 patients [ 30 ]. In addition, Al-Hatami et al. reported five candidemia cases co-infected with COVID-19 at a single center in Oman [ 31 ]. Furthermore, the results of a study conducted by Arastehfar et al., showed that among 1988 Iranian patients with COVID-19, seven had fungemia (7/1988; 0.03%) [ 13 ]. A study conducted by Chowdhary et al. indicated that candidemia affected 15 Indian coronavirus disease patients [ 32 ].

Furthermore, the majority of the patients with candidemia (9 of 12) in the current study were ≥ 50 years of age and our data showed that candidemia is more probable to infect older adult COVID-19 patients. Similarly, the results of a previous study showed the median age of the patients was 68 years (range 27–75 years) and concluded that older age is an important risk factor related to death in patients with COVID-19-associated candidemia [ 13 ].

In addition, the results of a study conducted by Bishburg et al. showed that the median age of the patients was 63 years [ 30 ]. During our experiences in the management of *Candida* bloodstream infections among patients with COVID‐19, we found that females were more affected than males by candidemia. Contrary to our results, several studies reported that men are infected with *Candida* bloodstream infections more frequently than women [ 31 - 33 ].

Regarding the severity of COVID-19, management methods, and clinical course of the disease, patients with severe COVID-19 infection are inescapably exposed to risk factors for developing opportunistic infections of *Candida* species and subsequently candidemia; including ICU admission, ventilation, corticosteroid administration, lymphocytopenia, receiving broad-spectrum antibiotics, and prior immunocompromised conditions. 

In our study, pneumonia (100%), lymphocytopenia (100%), the presence of the central venous catheter (100%), broad-spectrum antibiotic therapy (100%), mechanical ventilation (100%), and diabetes mellitus (50%) were observed as the risk factors for developing candidemia in COVID-19 patients. Diabetes mellitus was one of the most related risk factors predisposing COVID-19 patients to candidemia, with a prevalence of 50% in our study group.

Several studies have demonstrated higher rates of COVID-19-associated candidemia among people with diabetes in comparison to people without diabetes [ 32 , 33 ]. This is because diabetic patients are prone to high blood sugar concentration which weakens the immune system; hence, they are susceptible to fungal infections. [ 34 ]. Moreover, the results of the present study indicated that the broad-spectrum antibiotic therapy before candidemia was another risk factor associated with the occurrence of this bloodstream infection. In a study from Chinese hospitals, the administration rate of antibiotics and antifungal agents in patients with severe COVID-19 was reported 100% and 39 %, respectively [ 35 ].

The latest version of the Chinese clinical guideline for COVID-19 diagnosis and treatment recommends avoiding inappropriate use of antibacterial drugs, particularly the broad-spectrum ones, but without clear explicitness for empirical antibiotic therapy. This suggests that the administration of antibiotics on suspected COVID-19 patients is relying on the decision and experience of frontline clinicians, especially at the early stage of the pandemic outbreak [ 36 ].

It is worth mentioning that lymphocyte counts observed in 63-85% of patients with COVID-19 in literature were below the normal range, suggesting the lymphocytopenia as the main laboratory finding in COVID-19 infections [ 20 , 37 ]. The probable underlying cause of lymphocytopenia and subsequent susceptibility to candidemia in COVID-19 patients could be due to lymphocytes consumption by the virus. Notably, the decrease in T lymphocytes in HIV and SARS-CoV causes immunocompromised status in patients [ 38 ].

The presence of the central venous catheter at the onset of candidemia was another risk factor associated with the occurrence of candidemia in the present study. Accordingly, in previous studies [ 13 , 30 - 33 ], catheter retention was found as the main variable associated with increased risk of candidemia among COVID-19 patients. Although beneficial, venous catheters can predispose the patients to local infections of fungal origin, venous inflammations, and consequently spread of the fungal infection [ 39 ].

According to prior studies, ICU admission rate and invasive ventilation rate in COVID-19 patients were 16% and 8.3%, respectively [ 37 ]. Du and coworkers reported evidence of superinfections in 39% of COVID-19 patients hospitalized in China [ 35 ]. Several studies [ 13 , 31 , 33 ] revealed *Candida albicans* as the most common cause of candidemia in COVID-19 patients. Accordingly, *Candida albicans* was the predominant etiologic agent of candidemia in the present investigation. 

Furthermore, our findings showed that *Candida dubliniensis* was the causative agent of candidemia in two (16.6%) COVID-19 patients. *Candida dubliniensis* belongs to the oral microflora of healthy persons and its pathogenic role has been mainly restricted to oropharyngeal infections in AIDS patients [ 40 ]. However, some studies revealed that *Candida dubliniensis* can cause candidemia in immune-compromised patients [ 14 , 40 , 41 ]. Alataby et al. presented a case of a COVID-19 patient who developed *Candida dubliniensis* bloodstream infection and was treated with micafungin [ 42 ].

In this investigation, we reported the first two Iranian COVID-19 cases with candidemia due to *Candida dubliniensis* and the related antifungal susceptibility pattern. In general, in the current study, Amphotericin B and flucytosine were the most active drugs against *Candida* isolates recovered from blood cultures of Iranian COVID- 19 patients, and no case of resistance to these antifungal drugs was reported.

## Conclusion

In short, our data elucidated some concerns about the occurrence and management of candidemia due to *Candida albicans* and its related species *Candida dubliniensis* in Iranian COVID-19 patients. Data from our center could be a contribution to help decide on more effective strategies in antifungal treatments and to design a proper prophylaxis program for the benefit of such patients. However, this study had some limitations like the small sample size and the lack of previous research studies on the topic in Iran.

## Acknowledgement

This work was supported by funding from Tehran University of Medical Sciences, Tehran, Iran (Grant No: 1399-027). The authors wish to thank all the staff members of Imam Khomeini hospital, Tehran, Iran.

## Authors’ contribution

H.K.S., A. A, SJH, RDG, and Z.R. conceived of the study. H.K.S., S. Gh, P. AK, and S.A. performed the experiments. Z.R, Sh.M, and D.R prepared the manuscript. All authors read and approved the final manuscript. 

## Conflict of Interest

The authors have no conflicts of interest to declare for this study.

## Financial disclosure

No financial interests related to the material of this manuscript have been declared.
